# Survey of childhood diabetes and impact of school level educational interventions in rural schools in Karimnagar district

**DOI:** 10.4103/0973-3930.53123

**Published:** 2009

**Authors:** A. Avasarala Kameswararao, Anilkrishna Bachu

**Affiliations:** Department of Community Medicine and Epidemiology, Prathima Institute of Medical Sciences, Karimnagar - 505 417, AP, India

**Keywords:** Childhood diabetes, life style modifications, school health teams, Karimnagar district

## Abstract

**CONTEXT:**

Diabetes in children is increasing to epidemic proportions. It is essential to prevent its occurrence by screening for its modifiable risk factors at an earliest time.

**AIMS:**

1. To screen for childhood diabetes and its modifiable risk factors like obesity, physical inactivity, excessive eating of sweets, carbohydrate foods and chocolate intake and prolonged TV viewing. 2. To bring about reduction in these risk factors by lifestyle modifications through school health teams.

**SETTINGS AND DESIGN:**

A combined cross-sectional descriptive and interventional design among urban and rural school children was conducted.

**MATERIALS AND METHODS:**

Survey was conducted among 610 school children by 8 resident doctors to know the modifiable risk factors for diabetes. Four educational interventions to reduce and prevent the risk factors were carried out by the school health teams.

**STATISTICAL ANALYSIS USED:**

Proportions; χ^2^ test, t-test, cluster sampling.

**RESULTS:**

About 3.5% of children were diabetic. Reductions by 0.33%, 27.5%, 17%, 19% were achieved respectively in obesity, sweets/ chocolates/ carbohydrate rich food consumption, sedentary life and prolonged TV viewing.

**CONCLUSIONS:**

Childhood diabetes burden can be minimized by preventing the development of risk factors like obesity, sedentary life and eating excess of sweets, carbohydrate rich foods and chocolates. School level educational interventions through teacher-parent-child teams will definitely reduce the prevalence of diabetes.

## Introduction

Childhood diabetes is increasing rapidly globally,[1–6] including Asia.[2] WHO, in the year 1997, accepted it as major public health problem.[7] Once rare in children, now it has reached epidemic proportions,[1,2] and hence its prevention is urgent. Prevention can be done by life style modifications as it is a lifestyle related disease.[4] As it is childhood problem, school setting is ideal and convenient for conducting life style interventions.[8] Many people[6,9,10–12] conducted the risk factor interventions in schools. These types of interventional studies were rare in this part of the country.

## Materials and Methods

A two phased study (survey and intervention phases) was carried out in the year2007.

### Survey phase

In this phase, 610 school children from Rampur urban and Vutoor rural schools (Two clusters) were selected from Karimanagar district. Eight resident doctors (second author included) working at urban and rural health centres conducted the survey. A questionnaire containing the questions about variables like age sex, height (in cms) weight (in kgs), BMI for Age, socio-economic status of the family, literacy of the mother, occupation of the head of the family, physical activity, dietary history, TV viewing, outdoor playing, History of symptoms of diabetes was used for the survey. It was first pretested for deficiencies among 10% the sample of the children. The deficiencies were corrected and survey was continued. They examined and interviewed daily about 40 school children with the help of school teachers. To improve the validity of study, the information was obtained from all the school children in similar manner and for the similar duration. All the measurements, particularly, height and weight were recorded uniformly for all the children in a standardized manner and using the same instruments. The first phase of the survey was completed within 15 days.

### Case definitions considered for this study

- Childhood diabetes: Urine sugar estimation and glucose tolerance test using 75 gm glucose load was performed in the children to detect diabetic state. A child was considered diabetic if he was complaining of any or all of the symptoms of polyurea, polyphasia, polydypagia and abnormal glucose tolerance test with 75 grams glucose load. Urine sugar positive children without the positive glucose tolerance test were not considered as diabetics as they may be false positives.
- Daily TV viewing for more than 2 hours was considered as excessive.
- Excess sweets and sugars consumption was decided by the frequency and quantity of sugar foods consumed per week by the child. Weekly consumption of more than three times was considered as excessive consumption.
- Physical inactivity included children confined to indoors in the evenings, no outdoor play, not doing any physical exercises and not participating in games.
- Child hood obesity: The authors utilized better measurement for obesity i.e. BMI for age as it was more sensitive, highly specific and also gender specific for knowing the real magnitude of the childhood obesity.[5] It also has clinical and biological significance as children with BMI above 95^th^ percentile have a high probability of remaining obese and of experiencing the morbidity associated with obesity.[13] BMI-for-age was calculated using gender specific growth charts prepared by Centers for Disease Control and Prevention, Atlanta. They showed the percentile standards for body mass index, 2-20 years[14] wherein a cut off value of more than or equal to 95 percentile is used to define childhood obesity.[15] All the variables in the study population are analyzed in similar manner and significance of variables was calculated using proportions, χ^2^ and t-tests. Analysis was completed within a fortnight. All the preintervention data regarding the prevalence of diabetes in school children and its modifiable risk factors (obesity, excess sweets/ carbohydrate diet chocolates, sweets consumption, physical inactivity, prolonged TV viewing) was made ready for next phase of intervention.

### Interventions phase

It took another 15 days to contact the formal and informal leaders in the selected areas and to form four School Health Action teams. Each team led by two doctors comprised the children at risk, their parents and their class teacher and a local leader. Children were made into four groups depending upon the type of intervention they are in need of. These child groups included both the children at risk and not at risk and who will be benefited by the interventions. Each team was assigned one intervention to be completed in 6 months.

The four interventions assigned for four teams were:

1. Obesity prevention and reduction (OR team)
2. Prevention of excessive of sweets, chocolates and carbohydrateconsumption (SCC)
3. Reducing the duration of daily TV watching (TV team)
4. Increasing physical activity. (PAteam)

The teams were designated as obesity reduction team, SCC (sweets, chocolates and carbohydrates) team, PA team (physical activity) and TV teams. The resident doctors in charge of the teams prepared necessary and relevant health education materials. Each team conducted both theoretical and practical teaching sessions (interventions) using audiovisual aids at the rate of for 2 hours per week for six months i.e. 48 hours of motivation in total. Overhead projectors available at rural health centre were utilized for educating the teams and children. Here in the school curriculum, two hours per week were allocated for health care teaching. These hours were utilized for interventions. All the teams conducted the teaching sessions in the village and urban wards with help of local leaders and teachers. The idea is to disseminate this knowledge among entire community and increase their awareness of the problem. Follow up of the intervention was carried out by the teachers and doctors at weekly intervals.

All the obese children were educated regarding their risks for diabetes. They were advised to reduce the risk factors operating in them by means of physical exercise and diet modifications. Teachers, parent and community leaders were made aware of natural histories of childhood obesity, diabetes and their prevention and correction. Mothers and teachers are advised to encourage the children to play outdoor games and prevent them watching TV for longer periods. Mothers were advised to be very cautious while feeding their children. They were advised not to over feed their children with carbohydrate diet due to over -affection. Parents with a history of diabetes in their families were advised to be cautious while feeding their children. Urban rich families were advised to avoid feeding chocolates to their children.

Follow –up and evaluation: During the follow-up period the children were observed by the teachers and parents for life style changes. The impact of the interventions was assessed in three ways. Three different and separate impact questionnaires were used for parents, teachers and children to obtain their perspectives about impact. Pre- intervention and post- intervention results were analyzed. After the completion of this study, School health clinic functioning at urban and rural health centres, headed by pediatrician was made responsible for the sustenance of the benefits and effects on an ongoing basis. Every Thursday, the school health clinics were conducted in the health centres. The school children in the catchment areas of these centres were brought for counselling and treatment of childhood obesity. Obesity clinics were commenced where obese children are more.Diet plans, exercise plans, counselling and long term follow up sessions were carried out. Pediatric weight management programmes have been launched in these two areas.

## Results

1. Study population consisted 610 schoolchildren;
2. Prevalence of diabetes: 21 children (3.44%)
3. Overall prevalence of Childhood obesity: 59 children (9.67%) [Table 1]

**Table 1:** Urban – Rural and Sex distribution of obese children

Sex	Urban	Rural	Total
Boys	31 (5.08)	5 (0.81)	36 (5.9)
Girls	19 (3.11)	4 (0.66)	23 (3.77)
Total	50 (8.19)	9 (1.47)	59 (9.67)

Urban rural difference is not significant: X^2^= 0.13, *P*>0.05 (not significant)
4. Reductions by 0.33%, 27.5%, 17%, 19% were achieved respectively in obesity, sweets/ chocolates/ carbohydrate rich food consumption, sedentary life and prolonged TV watching. [Table 2]

**Table 2:** Showing the percentage of positive change in life styles after interventions

Targeted Interventions	Preintervention number of children (%)	Post intervention number of children (%)	Percentage of Net positive change
Obesity	59 (9.67)	57 (9.34)	0.33
Excess consumption of sweets, chocolates and carbohydrate rich foods	377 (61.8)	210 (34.4)	27.4
Prolonged daily TV watching	350 (57.3%)	232 (38)	19.3
Poor physical activity.	207 (33.9)	104 (17)	16.9

Difference is not significant: Paired t-test- t_df 3_ =2.81; *P*>0.05 (Not significant)

## Discussion

Prevalence of childhood diabetes revealed by this study is about 3.44%.

Plenty of studies[4,16,17,18–21] indicated high prevalence and increasing trends of childhood diabetes. Authors observed the risk factors like excessive intake of sugars {sweets, chocolates, and carbohydrate rich foods}, prolonged TV watching, physical inactivity, and obesity in that order in the study population. Every one believes that diabetes is due to eating too much of sweets and sugars. The similar opinion is revealed here (62%) and by Mohan *et al.* in his study.[20] Excessive TV viewing (57%) is the next offender detected here while it is about 73% in Urrutia study.[22] Physical inactivity is also considerable (34%) as seen with Renders[21] and Perez[23] studies. Last but not the least is the childhood obesity of about 10%. It is really high though less when compared to other contributing risk factors. Here the problem is method used for calculating the obesity[24–26] is body mass index alone which may not be realistic. If WHO[7] recommended body mass index of 21 is used, obesity will be more. If the body mass index of 30 and above is used, it will be less. Hence in this study, CDC, Atlanta's BMI for age and sex charts which are age and sex specific are used. This measurement is proper, better and realistic as growth is gender and age related. Some of the studies[17–20] linked the prevalence directly with obesity which is true here also.

Why the school children are selected for intervention? The reasons are obvious. School climate with its educators (teachers) is correct fit for any educational interventions and it is a child health problem. Petersen[8] also recommended schools as ideal settings for preventive programmes. School health teams are successful here in changing the negative life styles by their appropriate interventions. It is very unexpected and commendable to observe children reducing their sweets intake due to intervention. Net positive change is about **27%**. The habits of prolonged TV watching, physical inactivity are also considerably and favourably changed due to interventions. The most important change is reduction of obesity in two children within 6 months. Obesity is a tough risk factor to be tackled. That clearly indicates that the educational intervention is powerful and had a strong impact on children and parents. Here parents are very receptive as in Campbell study[10] in spite of their illiteracy. This clears that illiteracy cannot be a barrier when it comes to child health if the intervention is strong and powerful.

Positive strengths of this study are the easiest, feasible and necessary approaches that are utilized to achieve the objectives. Easiest in the sense, health educational motivational interventions which are simple, easiest, safest, and cost effective are used to bring about the change in negative lifestyles of children. These sessions increased awareness of the community at large, not only the selected children. This community sensitization will definitely have long term advantages to stop diabetes epidemic at large. Necessary in the sense, local leaders, teachers are necessary for the success, follow up and sustenance of the intervention. Hence they are actively involved by including them in action teams. It created interest in them and made them responsible for this health action. The idea of forming school health teams consisting of medical people, local leaders, teachers and parents and the beneficent children is encouraging. Making them responsible and accountable for the health of school children is a welcome feature in enhancing the quality of school health services. It is a successful community participation experiment. The positive gains of this study are undoubtedly due to active community participation to improve child health. Frankly speaking, neither the concept of school based interventions nor the community participation strategy are novel. But the intention to save the children from diabetes is good.

Limitations of the study: It must be remembered that primary community intervention trials, as such, are very difficult ventures. Enlisting and sustenance of community participation in a semi literate community and making a successful behavioural change, even a little, is worth appreciating. Though the interventions used are qualitative, diffuse and difficult to measure, maximum effort was made to make them and the study reliable. Physical inactivity is not considered in its true sense and three important things: frequency, intensity and duration are not measured. But it is assessed as an overall perspective in children.

Quality of school health services can be improved to reduce the burden of communicable and non – communicable diseases in children by forming and utilizing school health teams as done here. Only thing to be kept in mind, they have to be made responsible and accountable by proper policy changes.

## Conclusion

Life style changes among children with respect to childhood obesity, excess carbohydrate food consumption, excess sweets and chocolates consumption, physical inactivity and prolonged TV watching were introduced to an appreciable extent within six months through community participation. Obesity reduction and life style modifications in children as community programmes will go a long way in retarding or diminishing epidemic of diabetes. Particularly, the school level educational interventions combining both the behavioural programmes with nutritional programmes[27] through teacher-parent-child teams will be fruitful first steps in that direction.
